# Population study of thrombophilic markers and pharmacogenetic markers of warfarin prevalence in Bosnia and Herzegovina

**DOI:** 10.3325/cmj.2019.60.212

**Published:** 2019-06

**Authors:** Adna Ašić, Ramona Salazar, Niels Storm, Serkan Doğan, Wolfgang Höppner, Damir Marjanović, Dragan Primorac

**Affiliations:** 1 International Burch University, Genetics and Bioengineering, Sarajevo, Bosnia and Herzegovina; 2 Labor Dr. Heidrich und Kollegen MVZ GmbH, Molecular Genetics, Hamburg, Germany; 3 BioGlobe GmbH, Hamburg, Germany; 4 Institute for Anthropological Research, University of Zagreb, Zagreb, Croatia; 5 St. Catherine Specialty Hospital, Zabok/Zagreb, Croatia; 6 Eberly College of Science, The Pennsylvania State University, University Park, PA, USA; 7 School of Medicine, University of Split, Split, Croatia; 8 School of Medicine, Josip Juraj Strossmayer University of Osijek, Osijek, Croatia; 9 Faculty of Dental Medicine and Health Osijek, Josip Juraj Strossmayer University of Osijek, Osijek, Croatia; 10 Faculty of Medicine, University of Rijeka, Rijeka, Croatia; 11 Henry C. Lee College of Criminal Justice and Forensic Sciences, University of New Haven, West Haven, CT, USA; 12 Children’s Hospital Srebrnjak, Zagreb, Croatia

## Abstract

**Aim:**

To investigate the prevalence of common genetic variants that can serve as markers of thrombophilia and warfarin pharmacogenetics in Bosnia and Herzegovina.

**Methods:**

The study was performed between August and October 2017 on 130 healthy unrelated adult volunteers from Bosnian-Herzegovinian population sample. The prevalence of the following genetic variants was determined: *F5* c.1601G>A (factor V Leiden), *F2* c.*97G>A (factor II or prothrombin mutation), *F13A1* (factor XIII) c.103G>T, *MTHFR* (methylenetetrahydrofolate reductase) c.665C>T and c.1286A>C, as well as *PAI-1* (plasminogen activator inhibitor 1) c.-816A>G and c.-844G>A as markers of thrombophilia risk, and *2 and *3 alleles of *CYP2C9* (cytochrome P450 2C9) and five variants of *VKORC1* (vitamin K epoxide reductase complex subunit 1) as markers of warfarin pharmacogenetics. DNA was isolated from buccal swabs using salting out method, while genotyping was performed using matrix-assisted laser desorption/ionization-time-of-flight mass spectrometry.

**Results:**

Minor allele frequencies for two main thrombophilia risk factors, *F5* c.1601G>A and *F2* c.*97G>A were 0.023 and 0.008, respectively. Combined data for the markers of warfarin pharmacogenetics imply that 57.4% study participants can be expected to metabolize warfarin at an extensive, 40.3% at intermediate, and 2.3% at a poor rate.

**Conclusion:**

This study reports the first extensive population genetic data for thrombophilia and warfarin pharmacogenetic markers in Bosnia and Herzegovina. Allele frequencies of genetic variants are within the general average for European populations, and their presence implies the necessity of introduction of personalized medicine in warfarin-mediated antithrombotic therapy.

Thrombophilia is a condition conferring the risk of venous or arterial thrombosis and can be roughly classified into hereditary and acquired. Hereditary thrombophilia encompasses loss-of-function and gain-of-function genetic variants. Deregulation of coagulation cascade caused by loss-of-function variants, as well as their penetrance level, makes them more severe than gain-of-function variants, which are, however, around 10-fold more common in the general population (1,2). Currently, seven variants in five genes are routinely genotyped as genetic risk factors of inherited thrombophilia: *F5* c.1601G>A (factor V Leiden, FVL), *F2* c.*97G>A (factor II G20210A or *PTM,* prothrombin mutation), two *MTHFR* (methylenetetrahydrofolate reductase) variants, two *PAI-1* (plasminogen activator inhibitor 1) variants, and *F13A1* (factor XIII) c.103G>T (FXIII, V35L) (3-9). Warfarin, a coumarin derivative and vitamin K antagonist (VKA), is an oral anticoagulant taken to limit the production of coagulation factors II, VII, IX, and X. It is used for the prevention and treatment of thromboembolic events in patients with previous thromboembolism and atrial fibrillation (AF), following the implantation of artificial heart valves and vascular bypass procedures, as well as due to major orthopedic surgery (10-14).

Warfarin-mediated anticoagulation is currently considered to be a risky clinical therapy due to numerous interactions and interpatient variations in mean daily warfarin dose and narrow drug therapeutic range (10,12,13). We therefore hypothesized that a significant percentage of individuals from the general population will have minor variants in genes involved in thrombophilia risk, as well as warfarin pharmacodynamics and pharmacokinetics.

The aim of this study was to analyze the frequency of mutant alleles in two abovementioned sets of markers in Bosnia and Herzegovina (B&H). Additionally, we aimed to propose a simple and affordable method for genotyping relevant genetic variants that enables successful and safe implementation of personalized warfarin treatment in routine clinical practice.

## MATERIALS AND METHODS

### Sample collection and DNA isolation

Buccal swab samples were collected from 130 unrelated adult volunteers from B&H. Sample collection was performed in the medical laboratory Prolab (Sarajevo, B&H) between August and October 2017. Laboratory users were asked to participate in the study if they had no history of thrombotic events. In order to achieve a balanced distribution of participants’ demographic characteristics we included participants of both sexes, different age groups, and originating from different parts of the country as assessed by the origin of their grandparents. The size of the study population was determined according to previous population studies conducted in B&H (15,16) and was affected by financial constraints. Before agreeing to participate in the study, all participants signed the informed consent for study participation and data publication. Participants’ identity was known only to the main investigators. Ethical approval was obtained from the Ethics Committee of the International Burch University (Sarajevo, B&H) on July 6, 2017, document number 04-172-1/17. Genomic DNA was isolated from buccal swab samples using modified Miller’s protocol (17) at the International Burch University.

### Genotyping procedure

All genotyping procedures were performed in Bioglobe Laboratory, Hamburg, Germany. In order to analyze thrombophilia markers and pharmacogenetic markers of warfarin metabolism, two multiplexes and one uniplex iPLEX assays were set-up as follows:

1. Multiplex reaction for seven thrombophilia markers, including *F5* c.1601G>A, *F2* c.*97G>A, *MTHFR* c.665C>T and c.1286A>C, *PAI-1* c.-816A>G and c.-844G>A, and *F13A1* c.103G>T;

2. Multiplex reaction for six markers of warfarin pharmacogenetics: *VKORC1* (vitamin K epoxide reductase complex subunit 1) c.-1320G>A, *VKORC1* c.-1639G>A, *VKORC1* c.-679A>G, *VKORC1* c.174-136C>T, *VKORC1* c.283 + 124G>C and *CYP2C9* (cytochrome P450 2C9) c.1075A>C (CYP2C9*3);

3. Uniplex reaction for the marker of warfarin metabolism *CYP2C9* c.430C>T (CYP2C9*2).

Primer selection and plex design was performed in Assay Design Suite v2 (Agena Bioscience, San Diego, CA, USA). PCR reactions were set-up in 384-well plate with reaction components and thermal cycling conditions as previously described (18). In order to remove leftover dNTPs, shrimp alkaline phosphatase (SAP) treatment was employed as previously described (18). A general iPLEX reaction setup and cycling protocol was employed for all plexes using single-base extension chemistry and according to Storm et al (18) using 75 cycles. Thermal cycling was performed in GeneAmp PCR System 9700 (Applied Biosystems, Waltham, MA, USA).

Ion exchange sample conditioning was performed to remove excess cations from the sample-containing wells using Clean Resin (Agena Bioscience) (18). 15-nL samples were transferred to SpectroCHIP using MassARRAY Nanodispenser controlled by iPlex Genotyping for MassARRAY Nanodispenser software by Agena Bioscience. Matrix-assisted laser desorption/ionization-time-of-flight mass spectrometry measurement and genotyping of the products of iPLEX reactions were performed by using two software packages (both by Agena Bioscience): SpectroACQUIRE to obtain spectra and raw data and MassARRAY Typer to design sample plates and study assays, and access and export genotype data.

### Statistical analyses

Testing for Hardy-Weinberg equilibrium (HWE) was performed in GenAIEx v. 6.5 (*http://biology-assets.anu.edu.au/GenAlEx/Welcome.html*) using χ^2^ statistics at the significance level of 0.05 (19,20). Linkage disequilibrium (LD) testing was performed in a pairwise manner using the online version of GenePop v. 4.2 (*http://genepop.curtin.edu.au/)* at the significance level of 0.01 (21,22). Descriptive statistics for the study population and associations between demographic parameters and gene variants were calculated using IBM SPSS Statistics for Windows v. 23.2 (IBM Corp., Armonk, NY, USA), with Fisher exact test used at the significance level of 0.05.

## RESULTS

The group of elderly participants was underrepresented, with 6.2% sample donors being older than 60 years, since the majority of laboratory users who rejected to participate were from this age group (Table 1). Genotype data were successfully generated for all 130 participants for thrombophilia markers and for 129 participants for pharmacogenetic markers of warfarin metabolism.

**Table 1:** Demographic characteristics of study participants

Characteristic	n (%)
**Sex**	
**male**	47 (36.2)
**female**	83 (63.8)
**Age group (years)**	
**18-25**	54 (41.5)
**26-40**	44 (33.8)
**41-60**	24 (18.5)
**>60**	8 (6.2)
**Region**	
**Bosnian Krajina and Western Bosnia**	20 (15)
**Central Bosnia**	52 (40)
**Eastern Bosnia**	38 (29)
**Herzegovina**	20 (15)

Allele frequencies for two most significant markers of inherited thrombophilia were 0.023 and 0.008 for *F5* c.1601G>A and *F2* c.*97G>A, respectively. Neither age nor sex were significant predictors of variant thrombophilic marker distribution (Table 2) (Fisher exact test, *P* values for association with age: *P* = 0.645 for *F5* c.1601G>A, *P* = 0.840 for *MTHFR* c.665C>T, *P* = 0.160 for *PAI-1* c.-844G>A, and *P* > 0.999 for the remaining variants; *P* values for association with sex: *P* = 0.190 for *F5* c.1601G>A, *P* = 0.461 for *MTHFR* c.665C>T, *P* = 0.419 for *PAI-1* c.-816A>G, *P* = 0.533 for *F2* c.*97G>A, *P* = 0.366 for *MTHFR* c.1286A>C, *P* = 0.451 for *PAI-1* c.-844G>A, and *P* > 0.999 for *F13A1* c.103G>T). All study loci were in agreement with HWE (χ^2^ test, *P* > 0.05 for all seven loci). Using log likelihood ratio, two pairs of loci were found to be in strong linkage disequilibrium: 1) *MTHFR* c.665C>T and *MTHFR* c.1286A>C and 2) *PAI-1* c.-816A>G and *PAI-1* c.-844G>A (*P* < 0.01 in both cases).

**Table 2:** Genotype frequencies (with absolute genotype numbers in parentheses) and allele frequencies of risk markers of thrombophilia in Bosnian-Herzegovinian population. Allele frequency values are presented as major and minor allele frequencies*

Variant	Genotype frequency, % (n)			Allele frequency			
	major homozygous	heterozygous	minor homozygous	major		minor	
**FVL *(F5* c.1601G>A)**	95.4 (124)	4.6 (6)	0.0 (0)	**G**	0.977	**A**	0.023
***MTHFR* C677T (c.665C>T)**	43.8 (57)	46.2 (60)	10.0 (13)	**C**	0.669	**T**	0.331
***PAI-1* 4G/5G (c.-816A>G)**	30.0 (39)	50.8 (66)	19.2 (25)	**4G**	0.554	**5G**	0.446
FII G20210A (*F2* c.*97G>A)	98.5 (128)	1.5 (2)	0.0 (0)	**G**	0.992	**A**	0.008
***MTHFR* c.1286A>C**	46.2 (60)	43.1 (56)	10.8 (14)	**A**	0.677	**C**	0.323
***PAI-1* c.-844G>A**	15.4 (20)	51.5 (67)	33.1 (43)	**G**	0.412	**A**	0.588
***F13A1* c.103G>T (V35L)**	50.0 (65)	36.9 (48)	13.1 (17)	**G**	0.685	**T**	0.315

*F2, FII – factor II, *F5* – factor V, *F13A1* – factor XIII variant A1, FVL – factor V Leiden, *MTHFR* – methylenetetrahydrofolate reductase, *PAI-1* – plasminogen activator inhibitor 1.

Genotype data for markers of warfarin pharmacogenetics (Table 3) revealed that all polymorphic loci were in agreement with HWE (*P* > 0.05 for all five loci), while age and sex were not significantly associated with variant allele incidence (*P* values for association with age: *P* = 0.493 for *CYP2C9* c.430C>T and *P* > 0.999 for the remaining polymorphic loci; *P* values for association with sex: *P* = 0.294 for *CYP2C9* c.430C>T, *P* = 0.342 for *CYP2C9* c.1075A>C and *P* > 0.999 for the remaining three polymorphic loci). Three analyzed polymorphic *VKORC1* loci, c.-1639G>A, c.174-136C>T, and c.283 + 124G>C, were in complete linkage disequilibrium (*P* < 0.001). Statistical tests were employed only for the loci found to be polymorphic.

**Table 3:** Genotype frequencies (with absolute genotype numbers in parentheses) and allele frequencies of markers of warfarin pharmacogenetics in Bosnian-Herzegovinian population. Allele frequency values are presented as major and minor allele frequencies*

Variant	Genotype distribution, % (n)			Allele frequency			
	major homozygous	heterozygous	minor homozygous	major		minor	
CYP2C9*2 (c.430C>T)	74.4 (96)	24.8 (32)	0.8 (1)	**C**	0.868	**T**	0.132
CYP2C9*3 (c.1075A>C)	82.9 (107)	16.3 (21)	0.8 (1)	**A**	0.911	**C**	0.089
***VKORC1* c.-1639G>A**	39.5 (51)	45.7 (59)	14.7 (19)	**G**	0.624	**A**	0.376
***VKORC1* c.-1320G>A**	100.0 (129)	0.0 (0)	0.0 (0)	**G**	1.000	**A**	0.000
***VKORC1* c.-679A>G**	100.0 (129)	0.0 (0)	0.0 (0)	**A**	1.000	**G**	0.000
***VKORC1* c.174-136C>T**	39.5 (51)	45.7 (59)	14.7 (19)	**C**	0.624	**T**	0.376
***VKORC1* c.283 + 124G>C**	39.5 (51)	45.7 (59)	14.7 (19)	**G**	0.624	**C**	0.376

*CYP2C9 – cytochrome P450 2C9, *VKORC1* – vitamin K epoxide reductase complex subunit 1.

To determine the classes of warfarin metabolism according to metabolization rate (rapid, normal/extensive, intermediate or poor rate) (23), joint analysis of *CYP2C9* c.430C>T and c.1075A>C and *VKORC1* c.-1639G>A genotypes was performed (Table 4). A total of 57.4% study participants were classified as extensive metabolizers of warfarin. The rest of the participants were classified as warfarin-sensitive, that is, requiring daily warfarin dose decrease; 40.3% individuals had intermediate metabolism and 2.3% were poor warfarin metabolizers.

**Table 4:** Combined *VKORC1* c.-1639G>A and CYP2C9*2 and *3 genotypes in the study population from Bosnia and Herzegovina and genotype classification into extensive, intermediate, and poor metabolizer classes. The prediction of metabolizer class for each genotype was done according to Food and Drug Administration (FDA) recommendations from 2015 (23)*

*VKORC1* c.-1639G>A genotype	*CYP2C9* genotype	Number of participants (n)	Percentage distribution	Metabolizer class
GG	*1/*1	30	23.3	extensive
	*1/*2	11	8.5	extensive
	*1/*3	7	5.4	intermediate
	*2/*2	0	0.0	N/A
	*2/*3	3	2.3	intermediate
	*3/*3	0	0.0	N/A
GA	*1/*1	33	25.6	extensive
	*1/*2	15	11.6	intermediate
	*1/*3	9	7.0	intermediate
	*2/*2	1	0.8	intermediate
	*2/*3	0	0.0	N/A
	*3/*3	1	0.8	poor
AA	*1/*1	14	10.9	intermediate
	*1/*2	3	2.3	intermediate
	*1/*3	2	1.6	poor
	*2/*2	0	0.0	N/A
	*2/*3	0	0.0	N/A
	*3/*3	0	0.0	N/A

*CYP2C9 – cytochrome P450 2C9, *VKORC1* – vitamin K epoxide reductase complex subunit 1.

## DISCUSSION

Thrombophilia risk markers and genetic markers of warfarin pharmacogenetics were detected in a considerable proportion of general Bosnian-Herzegovinian population. The calculated allele frequencies are close to the previously reported European average.

The minor allele frequency of FVL prevalence in our study was 0.023. A previously reported FVL prevalence rate in healthy individuals in B&H was similar (Table 5). In Central Europe (Poland, Slovakia, Czech Republic), the frequency of mutant FVL was 0.039. In East Europe (Russia, Ukraine, Belarus) it was 0.019, while in South-Eastern Europe (Croatia, Serbia/Serbia and Montenegro, Macedonia, Slovenia, Bulgaria), it was 0.025 (30). A somewhat lower FVL prevalence was reported by the 1000 Genomes Project phase III data (Table 6) (31). MAF for *F2* c.*97G>A (*PTM*) of 0.008 in the present research is in agreement with previous studies in B&H and worldwide (Tables 5 and 6), in which minor allele was either the least abundant or absent from the population.

**Table 5:** Summary of previous research of thrombophilic marker prevalence in Bosnia and Herzegovina*

Study population	*N*	MAF	Major findings	Reference
Healthy B&H population	130	FVL 0.023 FII G20210A 0.008 *MTHFR* C677T 0.331 *MTHFR* c.1286A>C 0.323 *PAI-1* 4G/5G 0.446 *PAI-1* c.-844G>A 0.588 FXIIIA1 V35L 0.315	N/A	Current study
Healthy women	67	FVL 0.000	N/A	(24)
South-Eastern B&H healthy population	207	*MTHFR* C677T 0.333	N/A	(25)
Healthy adults	100	FVL 0.06 FII G20210A 0.06 *MTHFR* C677T 0.375	N/A	(26)
111 DVT patients and 207 healthy controls	318	FVL 0.0194 in controls and 0.105 in patients FII G20210A 0.000 in controls and 0.0136 in patients *MTHFR* C677T 0.2974 in controls and 0.3384 in patients	FVL variant was significantly more common in DVT patients than in controls	(27)
154 women who experienced PL and 154 female controls	308	FVL 0.039 in both controls and patients FII G20210A 0.016 in controls and 0.019 in patients *MTHFR* C677T 0.299 in controls and 0.357 in patients	none of three investigated markers was significantly correlated with the risk of PL	(28)
60 women who experienced RPL and 80 female controls	140	FVL 0.0188 in controls and 0.075 in patients FII G20210A 0.0063 in controls and 0.025 in patients *MTHFR* C677T 0.25 in controls and 0.3917 in patients *PAI-1* 4G/5G 0.2 in controls and 0.3 in patients	significant difference in allele distribution between the study groups for FVL and *MTHFR* C677T	(29)

*B&H – Bosnia and Herzegovina, DVT – deep vein thrombosis, FII – factor II, FVL – factor V Leiden, FXIIIA1 – factor XIII variant A1, MAF – minor allele frequency, *MTHFR* – methylenetetrahydrofolate reductase, *PAI-1* – plasminogen activator inhibitor 1, PL – pregnancy loss, RPL – recurrent pregnancy loss.

**Table 6:** The summary of the 1000 Genomes Project phase III findings (31). Minor allele frequency of six alleles in five global populations is given, namely African, admixed American, East Asian, European, and South Asian. Data for *PAI-1* 4G/5G were not available. Complete data set was retrieved from PharmGKB database (32)*

	African (n = 1322)	Admixed American (n = 694)	East Asian (n = 1008)	European (n = 1006)	South Asian (n = 978)
**FVL**	not detected	0.010	not detected	0.012	0.011
**FII G20210A**	not detected	0.014	not detected	0.008	not detected
***MTHFR* C677T**	0.090	0.474	0.296	0.365	0.119
***MTHFR* c. 1286A>C**	0.151	0.151	0.219	0.313	0.417
***PAI-1* c.-844G>A**	0.247	0.372	0.393	0.594	0.492
**FXIIIA1 V35L**	0.164	0.269	0.002	0.242	0.093

*FII – Factor II, FVL – Factor V Leiden, FXIIIA1 – factor XIII variant A1, *MTHFR* – methylenetetrahydrofolate reductase, *PAI-1* – plasminogen activator inhibitor 1.

*MTHFR* variants are globally most common in Europeans and Hispanics (Table 6), although heterozygous c.665C>T, homozygous c.1286A>C, or compound heterozygous genotypes currently seem not to have any clinical significance in thrombosis testing (9). *MTHFR* polymorphism is not included in the panel for inherited thrombophilia testing by the British Committee for Standards in Haematology and the British Society for Haematology. The American College of Medical Genetics and Genomics (ACMG) states that the informativeness of *MTHFR* testing is limited, and that homocysteine levels should be measured instead. In addition, ACMG recommends against *MTHFR* testing for the analysis of inherited thrombophilia, recurrent pregnancy loss, or for at-risk family members (9). c.665C>T variant has a prevalence of around 20% in Caucasians and Hispanics, 11% in Asians, and 1% in African-Americans (33). As regards *PAI-1* polymorphism c.-816A>G, also known as *PAI-1* 4G/5G, the present study reported 5G allele frequency of 0.446, which is significantly higher than previously reported (Table 5) (29). Marker *PAI-1* c.-844G>A had even higher prevalence in our population, with -844A allele having the frequency of 0.588, which is very close to the European average of 0.594 (Table 6).

Variant *F13A1* c.103G>T, also known as Val35Leu, has been assigned a protective role against venous thromboembolism, myocardial infarction (4,8,34), and coronary artery disease (35). MAF of 0.315 for this marker obtained in the present study is somewhat higher than previously reported T allele prevalence of 0.242 in Caucasians (Table 6).

Mutations in the latter three genes are considered to have additive value to other risk factors, including FVL and *PTM* mutations. Also, *PAI-1* variants were found to have a strong additive role in the increased incidence of myocardial infarction in the presence of metabolic syndrome risk factors, such as increased cholesterol and triglyceride levels (36).

*VKORC1* variant c.-1639G>A is one of the most extensively studied markers of warfarin pharmacodynamics. Its mutant allele A is associated with 2-fold lower gene expression and consequential lower warfarin requirements, increased risk of bleeding events, and international normalized ratio (INR)>4. The same is true for haplotypes A (warfarin-sensitive) and B (warfarin-resistant), consisting of c.-1639G>A and four additional loci, including c.174-136C>T and c.283 + 124G>C, inherited together due to extremely high level of linkage disequilibrium (37,38), which was proven in the current study as well.

When it comes to worldwide allele frequencies of c.-1639G>A, variant allele A is the most frequent in East Asians (0.86), followed by Europeans (0.37) and Africans (0.10). In US Hispanic population, MAF for this marker was 0.46 (39). The results from the current study for *VKORC1* variants are in alignment with previously published literature, especially two previous studies of *VKORC1* polymorphisms c.1173C>T and c.-1639G>A in Croatia. In the study by Mandić et al on 420 unrelated healthy individuals from Eastern Croatia, these two loci were in perfect linkage disequilibrium, with major and minor allele frequencies of 0.593 and 0.407, respectively (40). Another study analyzed the same two variants in 186 Croatian patients on stable warfarin therapy and found wild-type, heterozygous, and mutant homozygous genotypes frequencies to be 33.9%, 46.8%, and 19.4%, respectively (41).

*VKORC1* variants c.-1320G>A and c.-679A>G, together with missense change Asp36Tyr from exon 1 of the same gene, make up a haplotype that predisposes to high warfarin requirements, and these variants are more common in Jewish individuals than in other ethnic groups (42,43). However, these two loci were not polymorphic in general Bosnian-Herzegovinian population.

CYP2C9*2 allele (*CYP2C9* c.430C>T) was the first *CYP2C9* variant discovered and it stays the most studied SNP on this gene. Arg144Cys substitution encoded by this SNP disables a proper interaction of cytochrome P450 with NADPH-dependent cytochrome P450 oxidoreductase and decreases the rate of enzyme activity. Another important variant, *CYP2C9* c.1075A>C (CYP2C9*3), influences enzyme conformation, thus lowering its ability to bind different substrates, including warfarin (44). Daly et al report the global frequency of allele *2 of 0.0914, while allele *3 is less common, with an average worldwide frequency of 0.0637 (45). A previous population genetic study of *CYP2C9* polymorphisms in 200 unrelated individuals in Croatian population yielded results similar to ours. Alleles *2 and *3 had frequencies of 0.165 and 0.095, respectively (46). In the study of 129 healthy Slovenian individuals, alleles *2 and *3 had frequencies of 0.122 and 0.063, respectively (47). The only previous study on CYP2C9*2 allele in Bosnian-Herzegovinian population, which investigated the impact of this allele on the susceptibility to type 2 diabetes mellitus (T2DM) by genotyping 37 T2DM patients and 44 healthy controls, reported a low mutant allele frequency of 0.09) (48).

Novel or direct oral anticoagulants (NOACs, DOACs) are presently considered as an important improvement to traditional oral anticoagulation therapy, since they are less prone to interpatient variation, have stable pharmacokinetic and pharmacodynamic profiles exhibiting less interactions, and do not require frequent INR monitoring, thus decreasing or abandoning the need for warfarin dosing algorithms (13,49). European Society of Cardiology generally gives advantage to NOACs over VKA therapy for stroke prevention in AF (50) and treatment of deep vein thrombosis (51). While relevant data on NOAC usage in B&H are currently not available, the BALKAN-AF survey conducted in seven countries, including B&H, reported 10.1% of all patients on oral anticoagulation therapy to be taking NOACs prior to study enrollment. This number increased to 17.2% following the enrolling visit or hospitalization (52).

The limitations of the present study include the limited non-representative sample size of 130 individuals from the general population. This study thus does not offer information on the risk of thromboembolic events or recommendations for warfarin dosing at the population level. It is also important to note that this study alone does not provide guidelines regarding the duration or intensity of warfarin therapy in Bosnian-Herzegovinian patients. Future research should study the prevalence of the major genetic variants, including FVL, *F2* c.*97G>A *(PTM)*, *VKORC1* c.-1639G>A, and *CYP2C9* variants c.430C>T and c.1075A>C in a study group of warfarin-anticoagulated patients and a control group to assess their importance for personalization of antithrombotic treatment.

This is the first study reporting general population data for a panel of 14 genetic variants of inherited thrombophilia and warfarin pharmacogenetics in B&H. The results show that these genetic variants are considerably present in the general population of B&H. The frequency of detected minor variants is in agreement with previously published data for European populations. Implications of this study are important in that it paves the way for an integrated analysis of patient’s unique genomic makeup to offer individualized prophylaxis and treatment of thrombotic events.
